# Extending Kingdon’s Multiple Streams Policy Framework Through an Analysis of How Community Health Workers in India Are Driving Policy Changes

**DOI:** 10.1177/2752535X231222654

**Published:** 2023-12-17

**Authors:** Sanjana Santosh, Sumit Kane

**Affiliations:** 1 Centre for Health Policy and Systems, 29609 Gokhale Institute of Politics and Economics, Pune, India; 2 Melbourne School of Population and Global Health, The University of Melbourne, Melbourne, VIC, Australia

**Keywords:** community health workers, accredited social health activists, multiple streams framework, health policy change, COVID-19, India

## Abstract

In this paper we develop and provide a novel account of the process through which the Accredited Social Health Activists (ASHAs), a cadre of seemingly powerless community health workers in India, are navigating a complex policy process to incrementally achieve their goals. ASHAs have been demanding better working conditions, better compensation, and regularisation as public service employees through protests and strikes and have managed to gain concessions from both the Central and various State governments. We observed two important aspects that emerged: (a) ASHAs achieved incremental increases in their wages despite being the lowest in the health system hierarchy, and, (b) major gains were made during the 2 years of the pandemic. We examine and analyse ASHAs’ engagement and strategies used, both overt and covert, sometimes with the government, and the role of other actors in determining these policy outcomes. We do so by drawing on academic literature and news media reports; we trace the changes in ASHAs’ wages by tying together key events, ‘windows of opportunity’, and actions of ‘policy entrepreneurs’ involved in the process.

In doing so, we further develop and propose an extension to Kingdon’s multiple streams policy framework through the addition of a ‘narrative stream’.

## Introduction

In 2005, under the National Rural Health Mission (NRHM), India launched the Accredited Social Health Activists (ASHAs) program providing every Indian village with a female community health volunteer, the ‘ASHA’ or the Accredited Social Health Activist. ASHAs are all women; they are residents of the village they work in and are chosen and appointed by the village ‘Panchayat’ (the democratically elected village assembly). Selected from the village itself the ASHAs are trained to work as an interface between the community and the public health system. Over the last 15 years, the ASHA program has rapidly grown, and at a million strong, it is the largest community health worker (CHW) program in the world – it reaches every village in the country. ASHAs are volunteers who receive a modest, activity linked, honorarium. The NRHM provides the legal and administrative framework within which the ASHA is equipped with skills for providing care for a range of illnesses and is formally certified to do so. Studies show that many national disease-specific and other social welfare programs have increasingly called upon ASHAs to conduct and support activities in areas of maternal, new-born, child health, family planning, infectious diseases, and non-communicable diseases.^1–3^

## Institutional Arrangements and Working Conditions of Accredited Social Health Activists

**Figure 1.**
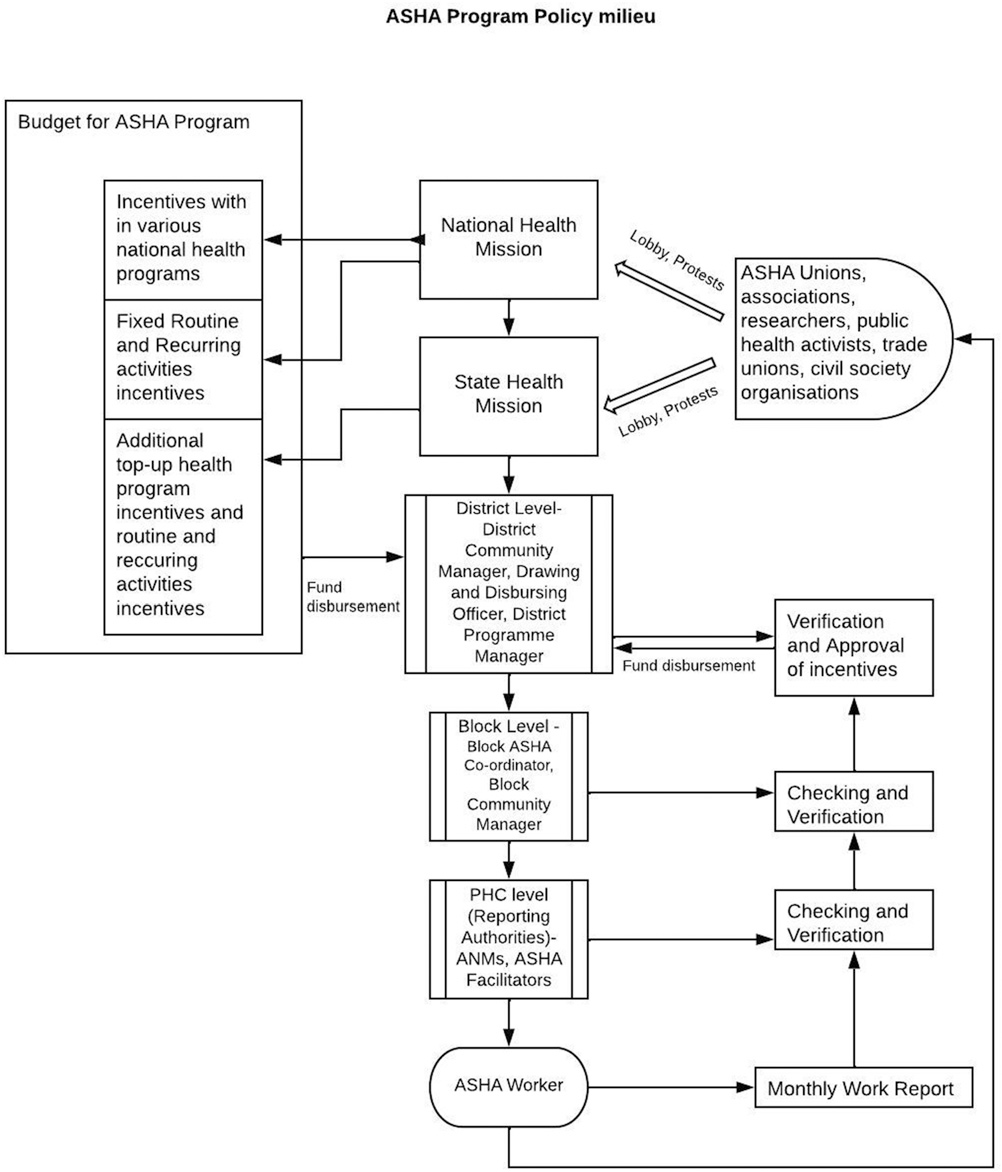
ASHA program policy milieu.

Originally, the ASHA position was conceptualised as an honorary volunteer without a fixed salary or honorarium and her work was supposed to be tailored in a way that does not interfere with her normal livelihood. She was to be compensated for the time and duration of her training. However, as the role of ASHA became prominent in various disease prevention programs a series of task-based incentives were introduced while maintaining the volunteer status. In addition to the 40 nationally approved task-based incentives, some states have also introduced fixed monthly honorarium or top-up incentives for ASHAs. The importance given to a particular health program depends on the context of the State and therefore the operations and incentives of a particular national program may differ among states. The incentives for ASHAs are thus drawn from both the Central or National budget as well as the State budget. Funds for making the payments to the ASHAs flow from the Centre to States and the States may add to these funds as per their discretion which then flows to the respective district administrations and then to primary health centres where the ASHAs are paid their incentives. (See Figure 1 below) Taking note of the expansion in the list of activities to be done by an ASHA, to ensure at least a minimum compensation for routine and recurring activities, an amount of INR 1000 per month was introduced by the Central government in 2013.

As the number of ASHAs have grown, and as their roles and tasks expanded, the ASHA program has attracted attention on multiple fronts - its design, its implementation, and its management arrangements and practices. How the program engages with the ASHAs, and the issues faced by ASHAs has attracted particular attention – from academics, civil society, and from public services too.^1,2^ The work conditions of the ASHA workers, especially their poor remuneration have been flagged in several studies. The delay in payments and lengthy procedures to process ASHA incentives have been documented in government documents as well as evaluations of the NRHM^3–5^ Lack of clarity about the pooling of incentives approved under different program budgets for ease of payments and delay in the release of funds from the state to the district level, emerged as the major bottlenecks for timely payments. We note these findings in the National Health Systems Resource Centre (NHSRC) report on the ASHA program in 2011 as well as in the NHSRC reports of 2017 and 2018. Some of the reports and studies also recommend changing the pattern of payment and highlighted the demands of a fixed honorarium or salary.^6,7^

Despite being the lowest in the health system hierarchy and despite their volunteer status and working conditions, the ASHAs have continued to grow in numbers and are increasingly the first point of contact for health services in rural areas. Some have argued that the increased responsibilities and involvement in various vertical health programs has led to a strong reliance of the health system on the ASHAs. Studies show that despite family pressures, despite dissatisfaction with working conditions, for many reasons^8,9^ ASHAs are rather keen on taking on more tasks and responsibilities.

For many years now, ASHAs have been demanding better working conditions, better compensation, and crucially asking for regularisation as public service employees. While ASHAs face other issues such as the onerous reporting requirements and workplace security, the demand for fair remuneration and formalisation as public servants has been an important rallying point and a policy priority. ASHAs have resorted to strikes, demonstrations, and *dharnas*^1^ to express their grievances and to register their claims in the broader social and political arena and have managed to gain concessions from both the Central and various State governments.

The Covid-19 pandemic added another layer of complexity by increasing the pressure on the health system, especially on front-line health workers, and aggravating the existing work conditions of the ASHAs. Several reports have highlighted the increase in workload, delay in payments, lack of clear training and instructions, and unavailability of masks and medical equipment.^10,11^ We note that the strikes and protests of ASHA workers have intensified in the 2 years (2020-2021) of the pandemic, and ASHAs and their supporters seem to be lobbying harder for their demands and negotiating in a more systematic manner with considerable success. In this process of lobbying, we observe two aspects that stand out - one, that ASHAs have been able to achieve these gains despite being at the bottom of the health system hierarchy, and second that major gains were made during the 2 years of the Covid-19 pandemic.

In this paper, we engage with these two aspects with a view to unpacking how ASHAs were able to make the gains they have made. We do so within the specific context of the State of Maharashtra. This contextual choice allows us, as health is primarily state-level matter in India, to specifically analyse the institutional and organisational conditions of the ASHA program, the structures of health policymaking, the specific actors involved, and the role of external events in influencing policy outcomes. We examine the status of ASHAs in the pre-covid and post-covid periods - we analyse ASHAs’ engagement with the government, the strategies they used, and the role of other actors involved in determining the policy outcomes. Analytically, we do this by taking the Covid-19 pandemic as an external ‘focusing’ event,^12^ through revealing the various major and minor ‘windows of opportunities’ (e.g., Guiraudon 2000: 260) exploited, through identifying ‘policy entrepreneurs’ (e.g., Tallberg 2003: 6) involved, and through locating the whole policy change exercise within the ‘national mood’ (e.g., Mansbridge 2003: 517).^13–15^ Our aim is to provide a novel explanatory account of the process through which ASHAs, a cadre of seemingly powerless health workers, are navigating a complex policy process to incrementally achieve their goals. To our knowledge, this is the first such account of a health worker cadre’s efforts to establish themselves as public service employees.

## Methodology

**Figure 2.**
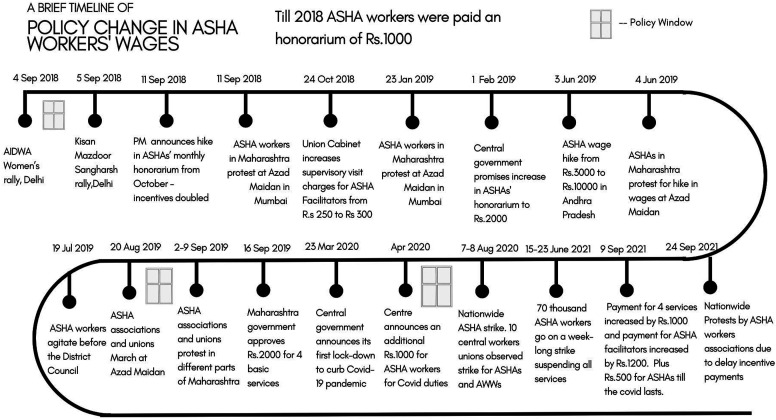
ASHA wages policy timeline.

We use secondary resources like government documents, media reports, evaluation reports and academic articles on the ASHA program to trace the recent policy change with respect to their work conditions, specifically in their wages. The government orders on ASHA workers status were acquired from the Maharashtra Government websites. For media reports, we used the Google web search with the filter of ‘news’ tab and sorted searches by ‘date’, ‘relevance’, and ‘hide duplicates’ tools available for advanced google search. We searched for news reports for the period of 1st August 2018 to 1st March 2020 and 1st April 2020 to 1st November 2021. The timeline was chosen based on important events around ASHAs work status and the beginning of Covid-19 pandemic. Google web search was a purposive choice given its expansive platform and the absence of physical newspapers during Covid-19 pandemic. News articles include reports from e-newspapers, online news platforms and popular news blogs. Only written forms of media were included in the study, other forms of media like videos were not included. The news reports were used to create a timeline of events and policy changes (Refer to Figure 2) and reviewed through directed content analysis.^16^ Broad codes were derived from the common themes emerging from the literature review on ASHAs work conditions in India and the state of Maharashtra and were used to analyse the articles using Atlas.ti software.

By drawing on various secondary resources we construct an account of the ASHA workers’ efforts to demand better payments and regularisation and how they use certain political moments as well as the Covid-19 pandemic as ‘policy windows’ to achieve policy gains. As we trace the developments in the ASHA workers’ conditions, we further discuss their issues as documented by several public health researchers and news media and note their role in providing vivid descriptions of the everyday life and profile of ASHAs. We broadly anchor our analysis in Kingdon’s Multiple Streams Framework (MSF) based understanding of the policy process.^17^ The application of Kingdon’s ‘policy window’ model to understand progress of health policy has been explored in health service research before using secondary resources and media analyses.^18,19^ We undertake a similar but more meticulous application of the MSF and while doing so we further develop and propose an extension to the MSF.

## Maharashtra Case Study

The state of Maharashtra currently has around 70,000 ASHA workers and 3500 ASHA facilitators working under the aegis of the State NRHM. Till 2018, the ASHA workers were paid a fixed honorarium of INR1000 for routine and recurring incentives by the Central government and other health program linked task-based incentives were provided by the Centre and the States according to the local contexts. The timeline below in Figure 2 shows how, over time, there have been various attempts by ASHA workers in certain time periods that were more conducive for lobbying, helping them achieve incremental changes. The timeline presents the evolution of the changes in response to ASHA’s demands of remuneration, regularisation and compensation, both at the national level and specifically in Maharashtra State. It traces both the pre and the post Covid-19 periods showing this evolution; it attempts to reveal how Covid-19 can be seen as a focusing event.

### Policy Change - Timeline

A quick glance at the timeline above (Figure 2) shows us that between September 2018 and September 2021, in most of the protests, ASHAs have demanded better incentives and a fixed salary. The Central and the Maharashtra State government have responded to these protests by increasing incentives marginally from time to time. While we find other demands alongside remuneration related demands, the emphasis of ASHA associations and unions has been on prioritising ‘ASHAs being unfairly underpaid’ as the primary policy problem. The policy solutions or demands pushed by the ASHAs, sometimes on their own, and sometimes in collaboration with other workers’ unions have remained consistent before and after the arrival of the pandemic.

We start tracing the timeline from the 4^th^ of September 2018 as it marks an important mass protest and rally where women’s organisations from across the country came together to demand rights for women in the national capital, New Delhi. This rally was organised by the All India Democratic Women's Association (AIDWA) primarily to hold the government accountable for its promises on women’s safety and security and it came after several incidents of violence against women.^20^ The declaration at the end of the rally included a range of demands for better employment rights and social welfare schemes for women; it also featured the demand for formalisation and regularisation of ASHAs as public service employees and increasing their salaries. There was another mass rally on 5th of September 2018, the very next day also in Delhi, the ‘Kisan Mazdoor Sangharsh’ rally, organised by the Communist Party of India -Marxist, the All-India Agricultural Workers Union (AIAWU), the Centre of Indian Trade Unions (CITU) and the All-India Kisan Sabha (AIKS) to bring attention to the plight of agricultural and non-agricultural labourers. Protestors from the AIDWA rally stayed over and joined the ‘Kisan Mazdoor Sangharsh’ rally – making common cause with agricultural and non-agricultural labourers and their unions. According to some news reports, for the first time in India’s recent history, the three main classes of workers - non-agricultural workers, farmers, and agricultural labourers - came together to rally around common causes.^21^ At both the AIDWA rally as well as the ‘Kisan-Mazdoor Sangharsh’ rally ASHAs demanded that their wages be increased to a minimum of Rs 18,000 per month. We see this demand for regularisation and a minimum wage of INR18,000 being consistently raised across multiple states in the time that followed.

These two rallies and the demands raised therein may have caught the attention of the political establishment and government administrators - as within a week of these protests, ASHA and Anganwadi workers across states received a public audience with Prime Minister Narendra Modi. This conversation between ASHAs and the Prime Minister was telecast live on national television as ‘Direct Samvad’ on the 11^th^ of September 2018. During this live interaction, Prime Minister (PM) announced a hike in ASHA workers’ routine and recurring activity incentives.^22^ This was called an ‘ASHA benefit package’ and included the revision of incentives for routine and recurring activities from INR.1000 per month (as revised in 2013) to INR2000 per month.^2^ The updated list of monthly ASHA incentives that emerged is presented in Table 1.

**Table 1.** Updated ASHA Incentives for Routine and Recurring Activities.

Incentives for routine and recurring activities	2013	2018
Mobilizing and attending VHND or (outreach session)	INR 200	INR 200
Convening and guiding monthly meetings of VHSNC/MAS	INR 150	INR 150
Attending a monthly meeting at Block PHC/5U-PHC	INR150	INR 150
∘ Line listing of households done at beginning of the year and updated every 6 months ∘ Maintaining records as per the desired norms like village health register ∘ Preparation of due list of children to be immunized updated on monthly basis ∘ Preparation of due list of ANC beneficiaries to be updated on monthly basis ∘ Preparation of list of eligible couples updated on monthly basis	INR500	INR1500
Total	INR1000	INR.2000

The ‘benefit package’ announced by the Prime Minister also included social security benefits like life insurance cover, accidental death or disability cover and pension for eligible ASHAs and ASHA Facilitators^3^. Fulfilling the promise made by the PM, the Central cabinet promptly (on 19th September 2018) approved an ordinance to increase routine and recurring incentives from INR1000 to INR2000 per month under the National Health Mission. Further on 25th October 2018, the Central cabinet increased the supervisory charges for ASHA facilitators from INR 250 to INR300 per visit taking note of the protests by ASHAs and ASHA facilitators.^23,24^

The announcement of the ‘ASHA benefit package’ by the Central Government sparked follow-on protests by ASHAs in multiple states asking State Governments take note of this development and demanding higher incentives and more importantly, a fixed salary – a demand which neither the Centre nor any of the State governments had yet to relent on. On September 11^th^, 2018, the ASHA unions staged a protest rally at Azad Maidan, Mumbai city-the state capital, demanding the Government of Maharashtra to increase their incentives and to provide a fixed salary to all ASHAs. Given that the incentives were paid from multiple program budgets and delay in processing incentives had been a constant issue, the increase in routine and recurring incentives by the Central Government did not enthuse ASHAs as it did not consider their demand for fixed income. In this whole process what stands out is how ASHAs strategically convey their demands from time to time to the Central as well as the State governments recognizing that since the financial responsibility for the ASHA program lies with both the Central and State governments, there is room to make inroads by expressing demands on both fronts. We note that the timelines reveal that national decisions or decisions of the Central government often seemed to spark protests and action at the state level - like in the case of Maharashtra and many other states.

On 23 January 2019, another massive protest was organised in Azad Maidan, Mumbai by the Alliance for Defence of Health Services and Rights (‘Arogyaseva Sanrakshan va Hakkansathi Aaghadi') - a coalition of ASHA Unions, ASHA Facilitators, Medical Officers, Nurses, Pharmacists, and other health workers in Maharashtra State; this was done together with a network of civil society organisations called the Jan Arogya Abhiyan. The protest demanded both a rise in ASHA’s remuneration and “immediate actions to improve public health services”.^25^

Later, on 1st February 2019, the Central Finance Minister in his Interim Budget speech announced the national budget which included the revised incentives for routine and recurring activities to INR2000 as announced by Prime Minister Narendra Modi in September 2018.^26^ The window of opportunity created by the September 4th and 5th protests in 2018 seemed to have propelled the revision of these incentives, however, the announcement of this in the annual budget also indicated that this would be a final response to ASHAs’ demands at the Central level until another opportunity emerged. It was unlikely that there would be another revision or possible regularisation in the same year and therefore the opportunity window to garner better payment from the Central government was seemingly closing.

### State Legislative Elections as Windows of Opportunity

The tussle between the Central and the State governments to commit resources to the ASHA program financially is both a challenge and an opportunity for the ASHA workers as it offers a larger pool of policymakers and administrators to negotiate with and leverage multiple opportunities to achieve the desired policy change. The political contexts in each state are often diverse presenting varying opportunities for ASHAs. We note that 2019 was particularly fruitful for ASHAs as they successfully exploited the pre- State legislative election periods as ‘windows of opportunities’ to push their agenda in many states. ASHAs have organised themselves under the aegis of well-established and politically savvy labour unions that have a country-wide networked presence and therefore their policy negotiations have benefitted from the guidance of experienced labour unionists. The role and importance of this network becomes evident when one sees the successes in one state context informing ASHA’s actions and strategies in other states. For example, ASHA associations and unions in the State of Andhra Pradesh successfully used the state legislative elections as a minor policy window when Chief Minister of Andhra Pradesh, YS Jagan Mohan Reddy on 3rd June 2019 announced an increase in the fixed wages of ASHAs (from Rs 3000 per month to Rs 10,000) fulfilling one of his pre-poll promises.^27^ A day after this announcement in Andhra Pradesh, on 4th June 2019, hundreds of ASHA workers in Maharashtra renewed their protests in Mumbai’s Azad Maidan for a similar increase in payment.^28^ This was organised by the same alliance - Alliance for Defence of Health Services and Rights which had organised the protest in January 2019. We see multiple protests organised by the ASHA workers in collaboration with other groups leading up to the State legislative elections in Maharashtra which was held in October 2019.

Protests were organised outside various District Councils across Maharashtra State on the 19^th^ July 2019 and then on 20th August 2019 protests were again organised in Mumbai. Closer to the elections, seemingly following the strategy of ASHAs from Andhra Pradesh, multiple protests were held (in Solapur and Kolhapur) in the 1st week of September 2019 which continued for eight consecutive days from 2nd to 9th amidst heavy rains. Similarly, a 3-days strike from 5-7th September 2019, was also organised in Aurangabad district.^29,30^ Responding to these protests and strikes, on the 16th of September 2019, the Maharashtra State Government, approved INR 2000 to be paid through the State budget for four routine and recurring activities by ASHAs.

### Covid-19 Pandemic as a Policy Window

**Figure 3.**
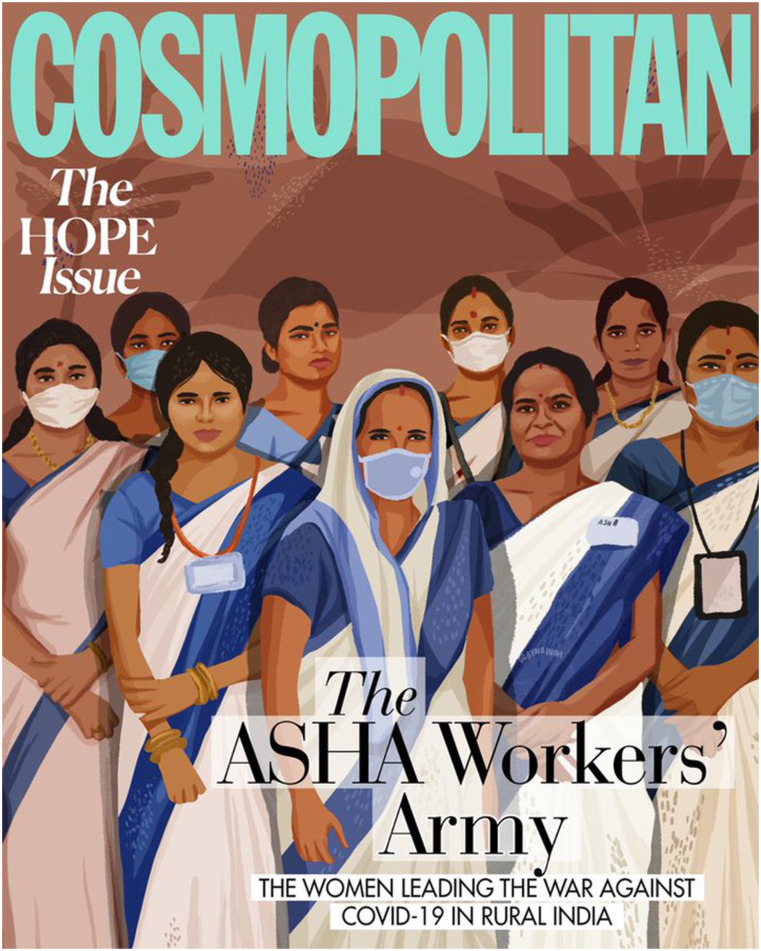
Cover page- Cosmopolitan- the hope issue: The ASHA workers’ army.

The Covid-19 pandemic hit India hard in 2020; the government imposed its first lockdown on 23rd March 2020. The pandemic put overwhelming pressure on the health system and health workers; it lent itself to be a ‘window of opportunity’ for health workers to lobby for their longstanding (and recent) demands as the public health crisis made them indispensable. The national (and global and local) focus on the health system and frontline health workers, not only activated ASHA associations and unions, the visibility of the issue in the public domain also activated many empathetic journalists, researchers, and public health activists who rallied around ASHAs’ cause. The reporting about ASHA’s plight in the mainstream media during this period was unprecedented. Even the upmarket COSMOPOLITAN magazine talked of the ‘ASHA Workers’ Army’ and dedicated its cover page to ASHAs (Figure 3)^31,32^

The pandemic affected all sections of the society, providing a common imagination around issues of social justice, health security and state welfare. The plight of wage workers, migrant workers, and workers in the informal sector was reported widely by the media – preparing a favourable milieu for ASHAs to push their agenda as wage workers and generate public sympathy. The narrative of ‘self-less and brave health workers’ was not only pushed by health activists and health workers associations but also by state administrators who sought public cooperation and health workers’ support for continuing health service provision amidst changing pandemic rules, guidelines and also to present themselves as competent leaders. For example, Prime Minister Narendra Modi called health workers ‘Corona Warriors’ urging the Indian public to clap and bang plates in the initial days of the lockdown in March 2020 and even organising showering of flower petals on district hospitals to show gratitude towards health workers.^33^ Throughout this period we see ASHAs strategically taking on this discursive mantle of ‘corona warriors’ to question the Central and the State governments and urging them to go beyond merely conferring honorifics and demanding for better health services, medical equipment, masks, sanitisers, PPE kits, and simultaneously pushing for increasing their remuneration. The Central Government, perhaps reading the public sentiment, announced an additional incentive of INR.1000 to all ASHAs for performing Covid-19 duties in April 2020.

It was however not always smooth sailing. For instance, in Maharashtra, a state with the highest number of Covid-19 cases in the country, although the previous government had promised and approved a hike of Rs.2000 on 16th September 2019, with the change in government post elections this promise was not fulfilled. After much wrangling by ASHA unions and associations, the Maharashtra Government, on the 24^th^ of June 2020 re-approved the previous promise of INR 2000 to ASHAs in addition to fixing salary for ASHA facilitators at INR 3000. News articles reported disappointment among ASHAs, and affiliated trade unions and ASHAs in several states including Maharashtra decided to stage a nationwide 2-days strike on 7th and 8th of August 2020.^34^ However, given the growing Covid-19 cases, the ASHAs in Maharashtra cancelled the strike and went to work on the 8th wearing black masks to express displeasure with the Government's decision.^35^ Even though the protest was called off, the support of 10 central trade unions in this nationwide strike demonstrated the ASHA workers’ ability to network, collaborate and mobilise themselves across states.^36^

The second wave of Covid-19 in India (around April 2021) was harsher and overwhelmed all parts of the health system. In rural areas this translated into greater demands being put on ASHAs as they were being called upon by the health system to serve without appropriate safety and security measures (Covid-19 related) in place. We see multiple meetings and many rounds of negotiations between ASHA workers and trade unions with the Maharashtra government in and around this period – with ASHAs demanding regularisation, and a minimum wage of INR 18,000 in June 2021. Following these meetings, the ASHA workers in Maharashtra began an indefinite strike and suspension of their services on the 15^th^ of June 2021.^37^ The growing interest and media attention on ASHA workers put immense pressure on the Maharashtra State government – it seemed to have forced them to increase the monthly incentives for routine and recurring activities of ASHA workers by a further INR 1000 and for ASHA facilitators by INR 1200. An additional INR 500 was also added as a Covid-19 incentive. This ended the indefinite strike in Maharashtra on 23rd June and the government of Maharashtra passed the order for this increase in incentives on 9th September 2021.^38^ Thus, during the pandemic, ASHAs in Maharashtra have gained a hike of INR 3000 (US$36.29) in their fixed honorarium for routine and recurring activities and INR 1000 and INR 500 (US$18.14) from the Central and Maharashtra State government as a covid incentive. ASHA facilitators have gained a hike of INR2200. Given the uncertainty of the pandemic, and new developments in the pandemic management, ASHAs launched another nationwide strike on 24th September 2021, this time protesting against delays in payment of incentives.^39^ It remains to be seen how far this policy window lends itself to gain more desired policy outcomes for ASHA workers in Maharashtra as well as at the national level.

## Discussion

### Setting the Policy Problem and Policy Solution-an Incremental Process

The ‘policy problem’ targeted by ASHAs - that of ‘poor payment and lack of formal employment’ was originally a design feature of the ASHAs. It evolved into a key concern as their role and duties expanded, and as ASHAs grew in number. The ‘policy change’ or demands pushed by the ASHAs, sometimes on their own, and sometimes in collaboration with other workers’ unions reflect this understanding of the ‘policy problem’ – and understanding which was at odds with those who designed the ASHA program and run it (the Central and State Governments, via the NRHM). Thus, as our analysis reveals, not only were ASHAs successful in putting their concerns on the policy agenda, crucially, they have successfully defined and established their version of the ‘policy problem’ as the version for all to engage with. This ownership and control over the agenda and over the definition of the issue is a major tactical win. It sets up the whole conversation and negotiation between ASHAs and the Government (s) to move in only one direction – even if the paths slightly differ across states, and even if the pace of progress changes at times.

This successful definition of the policy problem helped create a path dependency, and thus, optimal conditions for ASHAs to strategically choose the ‘policy solutions’ they wanted. Our findings suggest a very nuanced and very pragmatic approach here – a patient, tactical, and long-term play. While formalisation as public servants is the ultimate policy change that ASHAs are gunning for, they have approached this goal in a manner similar to a classic trade union tactic, as earlier sections show, negotiating consistently at every opportunity and settling for smaller and incremental gains.

ASHAs framed the policy demands and solutions and achieved incremental wins (policy changes) by strategically exploiting small and large windows of opportunities which both presented themselves and sometimes were created by them. ASHAs in fact tailored their approach to policy change (the policy solutions they pitched for and settled for) depending on the nature and size of the policy window. The nous with which ASHAs have navigated the tricky, but large policy window opened by Covid-19 is instructive and remarkable. While they have pushed relentlessly through the Covid period, they have also backed off when necessary – take for example, when the ASHA in Maharashtra resumed their work on the second day of the nationwide strike on 7th and 8th of August 2020 due to increasing Covid-19 numbers wearing symbolic black masks. Thus, striking a delicate balance between, keeping pressure on Central and State Governments, public sympathy, and maintaining support and least resistance from the public health service. ASHAs collaborating with established trade unions that have a nationwide presence and significant experience in organising labour rights campaigns, enabled both, inter-state coordination, and strategizing to optimally exploit windows of opportunity.

Our findings reveal how policy changes at specific political moments and especially during Covid-19, do not occur purely on the merit of the demands being made but depend on the convergence of enabling circumstances. Kingdon’s Multiple Streams Framework (MSF) is a heuristic to understand aspects of the policy process, particularly how certain ideas or issues gain policy attention and become part of policy agendas.^40^ Kingdon explains this by using aquatic metaphors arguing that problem identification and specification, policy solutions, and the politics around an issue are three streams that may flow separately but must come together during a brief ‘window of opportunity’ for policy change to occur. The narrative that traces the events around ASHA workers’ articulation of demands for hike in payments and regularisation of jobs clearly illustrates the consistency in the demands of ASHA workers before and during the pandemic.

For example, see below the news excerpts showing consistent framing of the ‘policy problem’ in Maharashtra pre and post covid around demands for increase in wages and formalisation of employment.

“A major focus of the protest is to demand a major raise in honorarium of ASHAs from current Rs 2,000 to the Rs 10,000 per month which is being given in other states such as Andhra Pradesh. The same alliance had organised a massive protest on January 23, 2019 at Azad Maidan in support of these demands, in response to which finance minister and Health minister promised some assurances, which are yet to be followed by even a single concrete action” (4th June 2019, Mumbai Mirror)^25^

“‘We are paid little and we work eight hours a day. Government is paying Rupees 33 per day for Covid surveys,' said Nashik-based ASHA worker Maya Bholap. Apart from Covid-related work, their duties include prenatal and postnatal care, immunisation drives for children, population-based screening for disease surveillance among others. Vinod Zodge from All India Trade Union Congress said they had demanded Rs 500 per day for Covid work. The entire workforce will go on a day’s strike on Tuesday and has decided to stop doing Covid duty from now.” (15th June 2021, The New Indian Express)^37^

The ‘policy solutions’ proposed by them arguably had been floating in what Kingdon would call the ‘policy primeval soup.’ Kingdon explains, in the ‘policy primeval soup’ policy solutions are proposed, considered, reconsidered, and modified by various actors involved before they are actualized or part of them gain policy attention^41^ The longstanding demands of ASHAs may have over time softened the government’s resolve^40^ (p. 116–117) – whether the acceptance by ASHAs of smaller, albeit significant concessions has created room for the big policy win of ‘regularisation’ remains to be seen. It is important to note that the policy solution stream also includes the strategies and tactics that the ‘policy entrepreneurs’ resort to, and which are also constantly reconsidered and modified in the ‘policy primeval soup.’ For example, the dual-track lobbying strategy that ASHAs use where they engage with both the Central and State level governments - was in play in the pre-Covid period and continued during the pandemic.

### Policy Entrepreneurs’ Role within Policy Windows

Kingdon argues that in the policy arena ‘policy entrepreneurs’ identify and frame problems, devise policy solutions, and apply tactics for the policymakers’ attention. The ‘policy entrepreneurs’ thus need to be alert to possible opportunities and to seize them whenever the political climate is suitable. The ‘politics stream’ refers to this political climate wherein policymakers may gain the motive to turn solutions into policy. This politics may involve certain political events, national mood, or changes in government ideologies, and it is the policy entrepreneurs’ onus to recognize the political climates and utilise the opportunities that it presents.^41^[p. 179–182] ASHA associations, unions and other trade unions supporting them are some of the active policy entrepreneurs we see protesting and lobbying for ASHAs demands at different times.

Let us look at ‘windows of opportunities' in the above timeline (Figure 2) to understand how the three streams of the policy problem, policy solution and politics couple and come together. To begin with, the first incremental policy gain comes right after the September 4th and 5th protests in 2018, wherein the window of opportunity is created by the coalition of trade unions, women’s organisations, ASHA unions and associations. This opportunity seems to be largely pushed open by a dynamic group of ‘policy entrepreneurs’ who successfully draw the attention of policymakers through sustained protests and by demonstrating large-scale mobilisation and collaborative strength. Even though the policy gain from these protests, the ‘ASHA benefit package’, was incremental, the ‘Direct Samvad’ event during which this was announced was an important political moment for ASHA workers - more of which we will unpack in the coming section.

The next window of opportunity in the timeline appears during the pre- State legislative elections wherein we see ASHAs in other states as well as in Maharashtra, by exploiting the political event of elections, address the government administrators’ motivation for re-election and to appear as benevolent leaders. We also see a diffusion of policy solutions or in this case policy strategies, wherein ASHAs in Maharashtra revived their protests a day after ASHAs in Andhra Pradesh managed to win INR 10,000 as a fixed compensation.^27^ Although the ASHAs in Maharashtra managed to win an incentive hike of INR 2000 before the State legislative election in September 2019, such pre-election, incremental policy changes are sometimes subject to reconsideration depending on the election result. The delay in the implementation of the promised hike for ASHAs in Maharashtra may be due to the change in government post-election. Elections, even though are recurring events and not ‘external’ or anomalous events, have the potential to yield policy gains based on the political context and the pre-elections mobilisation.

The diffusion of policy solutions and learning from other state mobilisations also raises questions about the nature of policy entrepreneurs and policy windows that are successful in certain state contexts and are not in others. While the differences among ASHAs across states are noted in some policy documents^7^ there is little literature that critically examines the issue of uniformity of ASHA’s work, status and wages across different states. We realise that we are constrained by our limited insight on what has occurred across the country and are drawing upon the specific case of Maharashtra. In terms of non-governmental actors or policy entrepreneurs, in our data we mostly encountered labour unions and cadre-based associations who were the key actors jostling for policy attention. We encountered a few instances where civil society organisations (CSOs) and networks were involved for example- organisations like Sathi-Cehat, networks like Alliance for Defence of Health Services and Rights, All India Democratic Women’s Association (AIDWA) and Jan Arogya Abhiyan in Maharashtra.^23,29^ We also see some involvement of CSOs in the work of narrative building through online articles.^42^ However, we did not encounter CSOs as prominent actors in the work of mobilising ASHAs in Maharashtra unlike trade unions and workers’ associations, perhaps due to the limitations of our chosen methodology. That said, the role of CSOs is an important one and their covert or overt role requires examination, as do the differences that exist across states. It is possible that owing to different working conditions and presence of varied policy entrepreneurs, ASHAs’ level of mobilisation, demands and policy wins differ across states despite similar recurring ‘windows of opportunities’ like the state legislative elections.

In contrast to a recurring window opportunity, the Covid-19 pandemic aligns with the description of the ‘external and unpredictable’ window allowing the ‘politics’ stream to dominate the process and the policy entrepreneurs to strategize and push for their policy solutions.^43^ The Covid-19 pandemic put the spotlight on the health system and health workers making the general public interested in how they will be provided care and by whom? In the next section, we explain how the Covid-19 pandemic narrative produced around health workers, especially ASHAs, allowed the ‘policy entrepreneurs’ engaged in their cause to grab policy attention.^44,45^

### The Centrality of Powerful Popular Narratives: Extending Kingdon’s Multiple Streams Framework (MSF)

Kingdon’s Multiple Streams Approach has been widely used and researched in policy studies; however, in our analysis of ASHAs' efforts to change policy, two points stand out and require unpacking. The extant literature on Kingdon’s MSF typically examines the coupling of the problem, policy, politics streams and the opening and closure of a policy window in question.^46^ While Kingdon has talked of policy windows as one-off events and that are often unpredictable, they may open multiple times, sometimes with great predictability and regularly.^17^(p. 184–186) To our knowledge, there are very few instances in the healthcare literature whereby the repeated coming together of the problem, policy, politics streams, and the repeated opening of small and large policy windows have been reported and well examined.^18,19^

**Figure 4.**
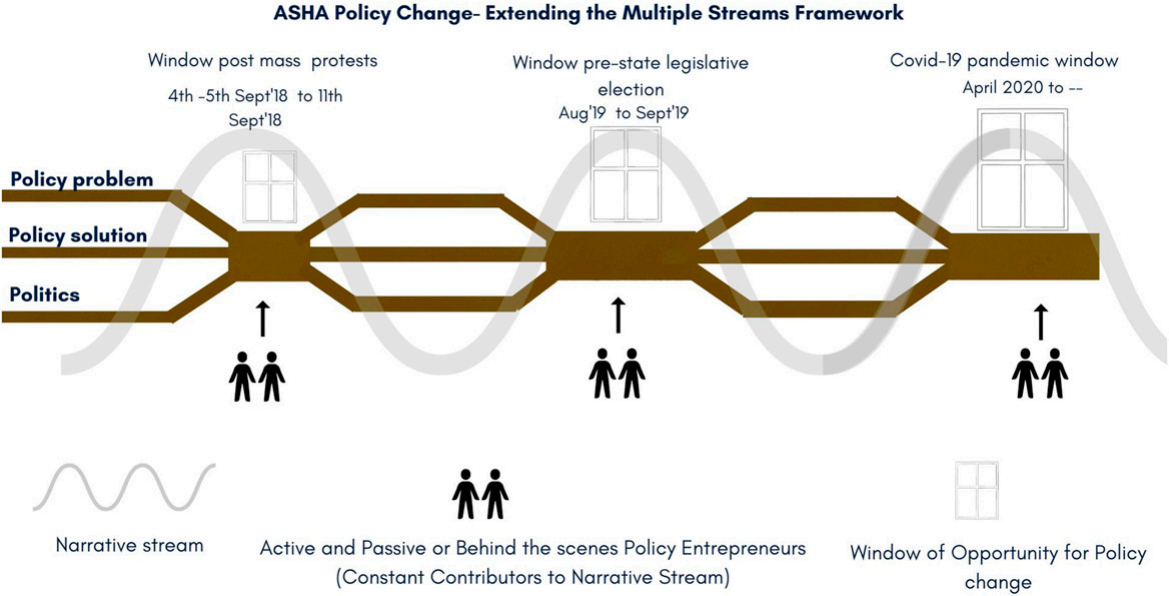
ASHA’s policy change – extending the multiple streams framework.

In Kingdon’s approach the ‘political stream’ encompasses politics and public opinion which gets stirred during a ‘focusing event’ that brings an agenda to attention and the actors advocate and lobby to create the politics required for desired policy change.^12,46^ Scholars have argued that although at times surprising and novel ‘focusing events’, in our case the pandemic, seldom activate new policy solutions as they are often pre-existing. In fact, such events activate existing politics around a particular issue and policy entrepreneurs choose the pre-existing policy solutions floating in the ‘primeval soup’.^46,47^ Interestingly, as we traced how ASHAs established their version of the ‘policy problem’ and ‘policy solution’, we also observed a consistent narrative about ASHAs being actively built, creating a favourable ‘politics’ for their desired policy outcomes. This narrative built over time, we contend, warrants separate attention than subsuming it within the ‘political stream’, as it appears to be cutting across the three streams extending the aquatic metaphor further by presenting itself as a separate *‘narrative stream’* (See below Figure 4).

As a sociological concept, narrative is a process by which people construct and communicate their understandings of the world. A narrative relies on selective use of past events ordered temporally and specific characters, and the events and characters must be related to one another and to an overarching structure. The positivist approach in social science research considered narratives to be ambiguous and too subjective to either be the object or method for sociological inquiry.^48^ However, ‘narratives’ eventually gained much attention as they allowed the bridging of daily social interaction and large-scale social structures and also due to its two important claims. First, from an epistemological perspective narratives were able to reveal truths about the social world that were obscured. Second, its political potential to give and preserve the voice of marginalised and silenced subjects.^49^ Postpositivist study approaches using narratives to explain its role in policy change have also received similar positivist criticism within the policy studies scholarship producing much debate on the need for structure and definition to examine narratives in policy studies.^50^ The literature on Narrative Policy Framework (NPF) acknowledges this debate and introduces a framework for thorough examination of the role of narratives and how they influence and shape public policies^51^ This serious treatment of narratives within policy studies allows researchers to focus on what exists objectively, like the policy actors and institutions, as well as on the important aspect of what people believe about the objects in the public policy arena. Furthermore, NPF literature provides an approach and framework to examine ‘subjective’ aspects like people’s emotions and foregrounds the role of narratives in human cognition, communication and decision making.^52^ While Kingdon’s framework has been used extensively to analyse health policy, the role of narratives has been overlooked. Public health policy researchers can greatly benefit from drawing on the above literature which would aid in holistic health policy analysis.^53^ In the case of this paper, this literature validates our findings and furthers the need for incorporating a separate stream-the ‘narrative stream’ within the Kindgdon’s multiple streams framework.

News media, professional organisations, research communities, courts and other such institutions are often the arenas where narratives about social problems are framed, dramatised, and presented.^54,55^ In our analysis in the context of ASHA workers, we observe actors including media platforms, politicians, academics, trade unions, and ASHA associations actively taking part in the meaning making of ASHA’s conditions producing a consistent narrative pre and post pandemic. The narrative stream’s role as a bund to keep the three streams (the problem, policy solution and politics streams) flowing became apparent as it permeated across the three streams consolidating policy problems, validating policy solutions and aiding and rebuilding the politics around ASHA’s demands.

#### Building the ASHA Narrative

In this section we discuss the nature of the narrative built and used by ASHAs and their supporters and reflect upon how it is shaping small and large policy wins. Our analysis suggests, multiple actors (as mentioned above) played an important part in laying out the ASHAs’ struggles, presenting a narrative about them being brave, selfless, and marginalised, and portraying them and their demands as legitimate and deserving.^56^

The policy and academic interest in Community Health Workers (CHWs) has been rising consistently since the 2000s^57^ Various World Health Organization reports (2006, 2010, 2018) have elaborated on the importance of the role of CHWs in delivering primary healthcare^58,59^ and to meet universal healthcare provisions and global health development goals (MDGs and SDGs).^60,61^ Especially in the context of reducing child and maternal mortality, a growing base of evidence on the positive contributions of CHW programs in population health encouraged several international funding for research on CHWs, for example - USAID, Bill and Melinda Gates Foundation, World Health Organisation, and research consortia like REACHOUT^62–67^ Given the consistent presence of CHWs on the global agenda, India being a country with one of the largest CHW programmes also attracted much attention both nationally and internationally. This has resulted in exponential growth of academic literature on ASHAs working conditions,^68^ their effectiveness in specific disease contexts,^69,70^ their performance,^71^ community relationships^72^ and their role in the health system.^73,74^ During the pandemic, researchers were able to access ASHAs remotely and sometimes physically to further document their experiences generating another round of flurry in the literature on ASHAs.^75,76^ The literature specifically on ASHAs' working conditions and role in the health systems have a solid contribution in the narrative building where in their marginalised position, and demand for better wages and regularisation is validated.^77^

While academic research has a specific kind of reach and use in agenda setting and policy advocacy, news media platforms offer greater scope in presenting issues in a more palatable and dramatised fashion. ASHAs seemed to be the most written about among the health personnel cadres as we encountered considerable news articles on ASHAs before and during the pandemic. The following news excerpts portray and build a certain profile and narrative of the ASHAs both in Maharashtra and other states in India

“For more than six lakh villages in India where medical facilities aren't easily accessible, a silent army of 860,000 health care professionals are the only ray of hope. However, this ‘pink army’ is not happy. Dismally low salaries, erratic work schedule and long working hours are just some of the issues that these women face. Come hail or high water, they are always all set. It does not matter to them if it is scorching hot or it is freezing cold, they perform their duties. These ladies are your ASHA workers – a silent army of health care professionals who, very diligently, look after expecting mothers and newborns in rural India.” (23rd Sep 2019, Gaon Connection)^78,^^4^

The use of adjectives like ‘pink army’ ‘silent army’ and descriptions of ASHAs’ motivations to help women and them enduring ‘scorching hot’, ‘freezing cold’, ‘erratic work schedules’ and ‘poor salaries’ build a vivid picture of them as hard-working people in poor working conditions. Even though their motives vary, the narrative of a ‘brave and selfless’ ASHA has contributions from several fronts creating unassuming ‘policy entrepreneurs’ who push open policy windows for their` cause. For example, let us consider the ‘Direct Samvad’ event, a live interaction between Prime Minister (PM) Narendra Modi and community health workers. This event was telecast live on 11th September 2018 giving ASHAs a national platform and an audience with the head of the state and the public. The PM spoke to health workers candidly and remarked that the way Hindu gods have several arms as a mark of their strength and power, ASHAs and other health workers were his ‘arms’ that allowed him to reach the masses in the remote corners of the country and serve the public [4:15]. In saying this the PM here elevated the position of ASHAs as workers who are engaged in godly work that is important for the government and reiterated the narrative that ASHAs were doing selfless and morally superior work. Immediately after the event, the PM announced the hike in ASHA’s remuneration (as presented earlier, this was limited to routine and recurring incentives). The narratives presented by the PM, the ASHA associations and unions, and the media, all fall on the same continuum – a paradoxical, yet remarkable and rare coming together of agendas of parties on opposite sides of the negotiation table.

This moment poignantly highlights the dynamic nature of the policymaking process; it also illustrates the fluidity of actor identities and agendas in such processes. This moment in the ASHAs’ campaign to push their agenda suggests that ‘policy entrepreneurs’ are not necessarily only those who are actively engaged in protesting, advocating, and lobbying for the cause. While ASHA workers associations and unions, and trade unions are and were undoubtedly the ‘active policy entrepreneurs’, there were many other ‘passive’ entrepreneurs like politicians and legislators and ‘behind the scenes’ actors like media houses, researchers, and public health activists; with some performing the role for reasons of their own, and others only coming on stage for a cameo.

We see similar but more detailed descriptions of the struggles of ASHA published in major media outlets, and in some research reports during the Covid-19 pandemic.

“People are scared of them […] they either shoo them away or shut the door on their face. But ASHAs continue to perform their duties. They don’t deserve to be treated like this. Strict action should be taken against those who use violence,” says BV Vijayalakshmi, general secretary of the National Federation of ASHA Workers. “They are the actual warriors in this fight [against Covid-19]. In fact, they’re not even given insurance. Nothing has been given to ASHA workers who passed away. Even if something happens to their family members, the government should provide help. The additional incentives announced last year haven’t been given till now.” (26 July 2021, Forbes India)^79^

We also note a similar presentation of the ‘marginalised ASHA’ narrative by some ‘passive policy entrepreneurs’ during the pandemic. In fact, we contend that policymakers and politicians rather than participating in this image building in a reactionary way like they did in the pre-pandemic time, played a more active role in creating a national imagination in favour of ASHAs and other health workers by declaring them as ‘Covid Warriors’.^33^ For example, on May 23 2020, Maharashtra State’s Chief Minister praised the ‘Covid-19 pandemic warriors’ for waging a ''War Without Arms'' against the invisible virus for the safety of people.^42^ These gestures and verbal displays of appreciation and care, while criticised by some health activists and academics as tokenistic, were strategically used by ASHAs and their proxies as discursive resources to garner public empathy and as tactical resources in their negotiations with the state. We see the moniker ‘Corona Warrior’ being consistently mobilised by ASHAs and their supporters to hold the government accountable and to further their demands.^80^ Descriptions of lack of adequate equipment and safety measures for ASHAs during the pandemic gave their demands further legitimacy.^37^

As we trace the events and narratives around ASHAs, we also observe how ASHAs achieve incremental success despite being a ‘powerless’ cadre in the health system. This powerlessness of ASHAs, as explained before, stems from their lack of formal employment and their bottom-most position in the health system hierarchy. However, ASHAs, by collaborating with actors seasoned in advocating for labour rights and by being completely accessible and available to varied actors like government bodies, CSOs, journalists and even researchers, subvert their disadvantages. They do so by drawing and learning from the knowledge and strategies of their collaborators to articulate their demands and by using their accessibility to project their voice and build a favourable narrative on news media and academic platforms.

## Conclusion

Through this account of tracing ASHAs workers’ demands in the State of Maharashtra, we see that ASHAs despite being the bottom most in health hierarchy are managing to achieve significant policy changes by collaborating with and mobilising various policy actors, producing and maintaining consistent narratives, and by relentlessly pursuing small and large policy windows. We show how they manage to define their policy problem, policy solution, partake in politics in varied political moments and exploit predictable and unpredictable policy windows. Recognizing the various kinds of actors who overtly or covertly, explicitly, or tacitly contribute to bringing a policy problem to light is a crucial first step towards understanding their motivations to participate in policy change and to reveal the nuances of a complex policy change process. The ostensible agreement around the narrative that ASHA workers are ‘warriors’, ‘brave’ and ‘noble’ across the spectrum of policy actors, active and passive policy entrepreneurs, we contend is a unique achievement of the ASHAs. ASHAs have been able to keep the narrative stream flowing in both pre and post Covid-19 period, giving them the discursive moral high ground, and greater leverage to resist and demand favourable policy outcomes. Our analysis shows how the inclusion of the narrative stream to the multiple streams framework enhances its analytical potential.

## Supplemental Material

Supplemental Material - Extending Kingdon’s Multiple Streams Policy Framework Through an Analysis of How Community Health Workers in India Are Driving Policy Changes Supplemental Material for Extending Kingdon’s Multiple Streams Policy Framework Through an Analysis of How Community Health Workers in India Are Driving Policy Changes by Sanjana Santosh and Sumit Kane in Community Health Equity Research and Policy

## Notes

1. A ‘dharna’ is the term used to describe a process that involves the collective and public (in a public space) expression of grievances or demands in the social and political sphere. The ‘dharna’ is a well-established democratic practice with antecedents in the non-violent civil action during the Indian freedom struggle.

2. The National Health Mission sends a official order to government health department executives acknowledging the change in ASHA incentives from 2013 to 2018 on 28th September 2018 https://nhm.gov.in/New_Updates_2018/communization/Aasha/Orders_and_guidelines/Revised_ASHA_Benefit_Package_(routine_incentives).pdf

3. ASHA Facilitators are supervisors who monitor ASHAs and compile the data collected by them for a cluster of villages. An ASHA facilitator has around 10-12 ASHAs reporting to her.

4. ‘Pink Army’ refers to the pink colour of the uniform of the ASHAs in some states.
