# Supplementary material for: Extending Kingdon’s Multiple Streams Policy Framework Through an Analysis of How Community Health Workers in India Are Driving Policy Changes
Source: Community Health Equity Res Policy. 2023 Dec 17;45(3):245–60. doi: 10.1177/2752535X231222654 (PMC11863506; doi:10.1177/2752535X231222654)
Supplement: Supplemental Material - Extending Kingdon’s Multiple Streams Policy Framework Through an Analysis of How Community Health Workers in India Are Driving Policy Changes [file sj-pdf-1-qch-10.1177_2752535X231222654.pdf]

Title: Extending Kingdon’s multiple streams policy framework through an analysis of how community health workers in India are driving policy changes.

Supplementary File: Code Framework and Search Strategy

1. **<u>Code Framework</u>**

- Codes
    - Subcodes

Codes/Subcode	Description
● **Salary/Payment issues:**	This code will be used to highlight issues of delayed salaries, underpaid staff, and protests to demand more pay by ASHAs. There will be two sub-codes used along with the broader code.
○ Underpaid ASHA	To capture system-level poor pay, delays, etc
○ Change in pay scale and policies	Mobilizing around pay and increase in incentives will be highlighted under this code to see how many articles speak of changes done before or after covid19
● **Advocacy/Mobilization**	This code will capture news highlighting any protests, negotiations, strikes, pressure-building tactics, or interaction with authorities.

○ General Authorities	To highlight mentions of authorities in a broad sense like government, state etc, and capture how they are talked about.
○ Specific Authorities	To highlight any specific authorities (government, unions, individuals in power positions) being mentioned and how they are talked about
● **Life conditions of ASHA**	To capture the living conditions, socio-economic status of ASHAs, how they are described, which profile of ASHAs and interviewed more
● **Nature of work**	To capture descriptions of ASHA's work, which kind of work gets reported more and how are they presented

2. **<u>Search Strategy Document</u>**

Search terms for media reports

ASHA, protest, wage, Maharashtra;

ASHA workers, wage, Maharashtra;

Inclusion criteria

- News articles available online on ASHA workers for the period of August 2018 to March 2020 and April 2020 to November 2021 will be included
- News articles include e-newspapers, online news platforms and popular news blogs, and online news magazines.
- Only written form of media will be included in the study
- News article should have word count of more than 250-300 words

Exclusion criteria

- News videos, news anchor commentaries will not be included
- Academic and analytical articles, opinion pieces will not be included.
- Evaluatory reports will not be included
- News reported outside India
- News reported not directly or indirectly linked to Maharashtra ASHAs

Identification and Screening Process

- Search hits for 1st August 2018 to 1st March 2020 (19 months) = 325 results. We screened till 10 search pages as Google recommends the top 10 search page results to be most relevant. Each page contains 10 search results. 100 results were identified for screening.

  After screening title and content both - **38 news articles** were included in the final analysis.

- Search hits for 1st April 2020 to 1st November 2021 (19 months) = 513 results. We screened till 10 search pages as Google searches recommend the top 10 search page results to be most relevant. Each page contains 10 search results. 100 results were identified for screening.

  After screening title and content both **- 60 news articles** were included in the final analysis
